# The impact of delayed versus early administration of granulocyte colony-stimulating factor following autologous hematopoietic stem cell transplantation on transplantation outcome

**DOI:** 10.3389/fonc.2024.1468948

**Published:** 2024-10-01

**Authors:** Mahshid Mahdizadeh, Mohammad Amin Karimi, Zohreh Tajabadi, Vahid Kaveh, Shayan Zamani

**Affiliations:** ^1^ Hematopoietic Stem Cell Research Center, Shahid Beheshti University of Medical Sciences, Tehran, Iran; ^2^ Taleghani Hospital Clinical Research Development Unit, Shahid Beheshti University of Medical Sciences, Tehran, Iran; ^3^ School of Medicine, Shahid Beheshti University of Medical Sciences, Tehran, Iran; ^4^ Digestive Disease Research Institute, Tehran University of Medical Sciences, Tehran, Iran; ^5^ Hematology and Oncology Department, Iran University of Medical Sciences, Tehran, Iran; ^6^ Center for Research and Training in Skin Diseases and Leprosy, Tehran University of Medical Sciences, Tehran, Iran

**Keywords:** hematopoietic stem cell transplantation, granulocyte colony-stimulating factor, delayed administration, early administration, engraftment

## Abstract

**Objectives:**

Granulocyte colony-stimulating factor (G-CSF) is routinely administered after autologous hematopoietic stem cell transplantation (auto-HSCT) to decrease the duration of neutropenia and diminish the incidence of febrile neutropenia. Nevertheless, the most advantageous timeframe for administering G-CSF in the transplantation setting remains elusive.

**Material and Methods:**

We conducted a cross-sectional study of 200 patients diagnosed with hematological malignancies who underwent auto-HSCT between July 2017 and January 2022. Patients were divided into two groups of 100 individuals based on the timing of G-CSF administration after auto-HSCT. In the first group, G-CSF was administered on post-transplantation day +1, while in the second group, G-CSF was administered on post-transplantation day +5. Patient demographics and clinical outcomes, including time to neutrophil engraftment, time to platelet engraftment, length of hospital stay, duration of fever, and incidence of bacterial and fungal bloodstream infections, were compared between the two groups.

**Results:**

We identified a significantly shorter platelet engraftment time in the day +5 group than in the day +1 group (P<0.001), though the groups were similar regarding neutrophil engraftment time. The total number of G-CSF injections differed significantly according to the administration schedule. The number of red blood cells and length of hospital stay was greater in the day +1 group (all P<0.001). The incidence of bacterial and fungal bloodstream infections and duration of fever did not differ between the groups.

**Conclusion:**

Delayed administration of G-CSF on day +5 is as effective as early administration and can positively influence platelet engraftment, transfusion support, and hospitalization time.

## 1 Introduction

Hematopoietic stem cell transplantation is a widely recognized sequential process aimed at substituting an individual’s blood and lymphoid systems with a fresh system obtained from hematopoietic stem cells (HSCs). These HSCs can be procured from a healthy donor (referred to as allogeneic transplantation) or from the patient (known as autologous transplantation). Over the past six decades, this procedure has been extensively employed to treat aggressive hematological malignancies, including leukemia and lymphoma. Autologous hematopoietic stem cell transplantation (auto-HSCT) is a widely used therapeutic option in managing patients with hematological malignancies and non-malignant hematologic and genetic disorders (1, 2).

As part of transplant preparation, high-dose chemotherapy can cause neutropenia, potentiating severe and potentially fatal complications such as infections (3, 4). The course of neutrophil recovery following auto-HSCT is influenced partly by the exogenous administration of myeloid growth factors such as granulocyte colony-stimulating factors (G-CSFs) (5). G-CSF is a glycoprotein and hematopoietic cytokine that stimulates the mobilization and activation of neutrophils and their precursors, thus attenuating the severity and duration of neutropenia and its clinical complications (6). Current guidelines recommend that G-CSF be started 1–5 days after high-dose chemotherapy and auto-HSCT administration and continued until neutrophil recovery (7). Myeloid engraftment is the first of three consecutive days with an absolute neutrophil count (ANC) of 0.5 × 10^9^/L or more; growth factors usually continue until the ANC remains above this threshold for three consecutive days (8).

A growing body of interest is developing regarding the delayed initiation of G-CSF after auto-HSCT, mainly because of substantial economic benefits and drug side effects. Based on animal studies of bone marrow (BM) cell kinetics, most dividing myeloid progenitors/precursors are eliminated from the BM immediately after administering high-dose chemotherapy and do not reappear immediately after SC re-administration, making early G-CSF worthless (9). Studies that examined two practices of post-transplantation G-CSF starting points, early (day 0 to day +4) and late, reported inconsistent results regarding time to neutrophil and platelet engraftment, length of hospitalization, and incidence of febrile neutropenia (10–14). Most of these studies were conducted on small numbers of patients and varied significantly in patient characteristics and G-CSF regimen. More importantly, these studies had differences in the distribution of factors affecting transplant outcomes.

Overall, there needs to be a clear consensus about the optimum schedule of G-CSF administration post-transplant in clinical practice. This necessitates further evaluation to determine a reasonable starting point that would be clinically and financially beneficial, which was the aim of the present study. We hypothesized that delaying G-CSF administration to day +5 following auto-HSCT would achieve comparable or improved outcomes with reduced resource utilization compared to standard day +1 administration. We compared the auto-HSCT process between two groups of patients with different post-transplant G-CSF starting points in the Taleghani Bone Marrow Transplantation (BMT) center in Iran.

## 2 Patients and methods

### 2.1 Study design and participants

We performed a retrospective cross-sectional study to compare two schedules of G-CSF administration, early or delayed, during the post-transplant period. We evaluated the charts of 200 patients who underwent auto-HSCT for hematological malignancies or solid tumors and randomly received post-transplantation GCSF on day +1 or day +5 between July 2017 and January 2022 at Taleghani BMT Center. The Research Ethics Committee of Shahid Beheshti University of Medical Sciences (IR.SBMU.REC1390.568) approved the study protocol. Written informed consent was obtained from all patients. We divided the patients into two groups: 1) patients who received G-CSF (PDgrastim^®^, Pooyesh Darou, IR) 24 hours after the administration of the peripheral blood stem cells (day +1 group) and 2) patients who started G-CSF on the 5th day after transplantation (day +5 group). G-CSF at 5 µg/kg was intravenously administered in all cases and continued until day +10, and the dose was increased to 10 µg/kg until the ANC recovered to ≥ 0.5× 10^9^/L for three consecutive days.

### 2.2 Clinical protocol

In the setting of auto-HSCT, 10 µg/kg of G-CSF was given subcutaneously for five days to collect a transplantable dose of CD34+ cells (>2×10^6^ CD34+ cells/kg). Then, G-CSF-mobilized peripheral blood progenitor cells (PBPCs) were separated from the peripheral bloodstream using the Spectra Optia (Terumo BCT) apheresis machine system and measured by fluorescent-activated cell sorting. The underlying disease determined the typical preparative regimen for auto-HSCT in our hospital. It included a CEAM regimen (lomustine, etoposide, cytarabine, and melphalan) for patients with Hodgkin’s or non-Hodgkin’s lymphoma and high-dose melphalan for patients with myeloma. CD34+ cells were infused 12-24 hours after the last dose of chemotherapy (based on the drug’s half-life).

Based on our center’s protocols, patients with a hemoglobin (Hb) level below 7 g/dl received a blood transfusion, and if the platelet count was below 10,000, they received a platelet transfusion. Additionally, if the patient had a fever above 38.3°C or above 38°C on two consecutive measurements one hour apart, they were administered antibiotics.

### 2.3 Outcome variables

Data collected included patient demographics, clinical and laboratory results, and patient transplantation outcomes. Clinical and laboratory variables included body mass index (BMI), diagnosis, conditioning regimens, previous history of local radiotherapy, BM pathologic status, and remission status. Patient transplantation outcomes included time to platelet and neutrophil engraftment (defined as platelet count > 20×10^9^/L on three separate measurements without transfusion support or ANC > 0.5×10^9^/L on three individual measurements), transfusion requirements during transplantation episode, fever, the incidence of bacterial and fungal bloodstream infections, and length of hospital stay.

### 2.4 Statistical analysis

Data were managed using SPSS statistical software (version 20.0, USA). Categorical variables are summarized as frequencies and percentages. Continuous variables are expressed as mean values and standard deviation (SD) or median and interquartile range depending on the data’s distribution and normality. Chi-squared, Mann-Whitney U, and Kruskal-Wallis tests were used where appropriate to assess differences between groups. The log-rank test was used to compare probabilities of engraftments, fever duration, and length of hospital stay between the groups. A P-value of less than 0.05 was considered significant.

## 3 Results

### 3.1 Baseline characteristics of patients

The current study enrolled 200 patients with malignancies who underwent auto-HSCT from July 2017 to January 2022 at Taleghani BMT Center, Tehran, Iran. 114 (57%) patients were male, and 117 (58.5%) participants had complete remission at the time of transplantation. Among patients, 85 (42.5%) suffered from multiple myeloma, 73 (36.5%) from Hodgkin’s lymphoma, 35 (17.5%) from non-Hodgkin’s lymphoma, four (2%) from solid tumors, and three (1.5%) from acute myeloid leukemia. Regarding conditioning regimens, 110 (55%) received CEAM, 83 (41.5%) high-dose melphalan (200 mg/m^2^ in patients <55 years old and 140 mg/m^2^ in patients >55), three (1.5%) germ cell therapy (high dose ICE), three (1.5%) AML (melphalan busulfan), and one (0.5%) received the Ewing protocol. Notably, 67 (33.5%) participants had a history of radiation therapy. Regarding chemotherapy lines, 153 (76.5%) participants received less than two lines, mostly multiple myeloma patients (**Table 1**).

**Table 1:** Baseline characteristics of patients based on time of granulocyte colony-stimulating factor (GCSF) initiation.

Characteristic	Day +1 group (N=100)	Day +5 group (N=100)	P-value
**Gender, n (%)** **Female** **Male**	41 (41.0%) 59 (59.0%)	45 (45.0%) 55 (55.0%)	0.57
**Age, years (median, IQR)**	38.5 (25.25 – 51.0)	46.5 (29.0 – 52.0)	0.049
**BMI, kg/m^2^ (mean ± SD)**	26.97 (5.5)	26.6 (5.2)	0.66
**Underlying malignancy, n (%)** **Multiple myeloma** **Hodgkin’s lymphoma** **Non-Hodgkin’s lymphoma** **Solid tumor** **Acute myeloid leukemia**	35 (35.0%) 38 (38.0%) 23 (23.0%) 3 (3.0%) 1 (1.0%)	50 (50.0%) 35 (3.0%) 12 (12.0%) 1 (1.0%) 2 (2.0%)	0.086
**Conditioning regimens, n (%)** **CEAM^1^** **HD Melphalan^2^** **Bu-Mel^3^** **HD ICE^4^** **Bu-Mel ^5^**	61 (61.0%) 34 (34.0%) 0 (0.0%) 3 (3.0%) 1 (1.0%)	47 (47.0%) 49 (49.0%) 1 (1.0%) 0 (0.0%) 2 (2.0%)	0.026
**History of radiation therapy, n (%)**	37 (37.0%)	30 (30.0%)	0.37
**Chemotherapy line, n (%)** **<2** **≥2**	71 (71.0%) 29 (29.0%)	82 (82.0%) 18 (18.0%)	0.075
CD34^+^ cell count, *10^6^/kg (median, IQR) **Harvested CD34^+^ cell** **Infused CD34^+^ cell**	3.7 (1 – 4.7) 3.7 (1.2 – 4.9)	3.6 (1.0 – 4.6) 3.5 (1.0 – 4.5)	0.11 0.08
**Disease remission status** **Partial remission** **Complete remission**	38 (38.0%) 62 (62.0%)	45 (45.0%) 55 (55.0%)	0.31

BMI, body mass index; IQR, interquartile range; N, number; SD, standard deviation; CEAM, CCNU, Etoposide, Cytarabine, and Melphalan; MM, Multiple myeloma; HD, high dose; Bu-Mel, busulfan-Melphalan; ICE, Ifosfamide, Carboplatin and Etoposide.

^1^Regimen for patients with NHL Hodgkin’s disease; ^2^Regimen for patients with Multiple Myeloma; ^3^Regimen for patients with Ewing sarcoma; ^4^Regimen for patients with Germ Cell Tumors; ^5^Regimen for patients with Acute Myeloid Leukemia.

The baseline characteristics of participants before auto-HSCT are listed in **Table 1**. Statistical analysis revealed no significant difference regarding gender, BMI, underlying malignancy, history of radiotherapy, chemotherapy lines, disease clinical remission status, and CD34^+^ cell count between patients who received GCSF one day after auto-HSCT and those who received GCSF five days after transplantation (*P*>0.05). However, some significant differences were seen in age and conditioning regimens (**Table 1**).

### 3.2 Clinical outcomes

**Figure 1:**
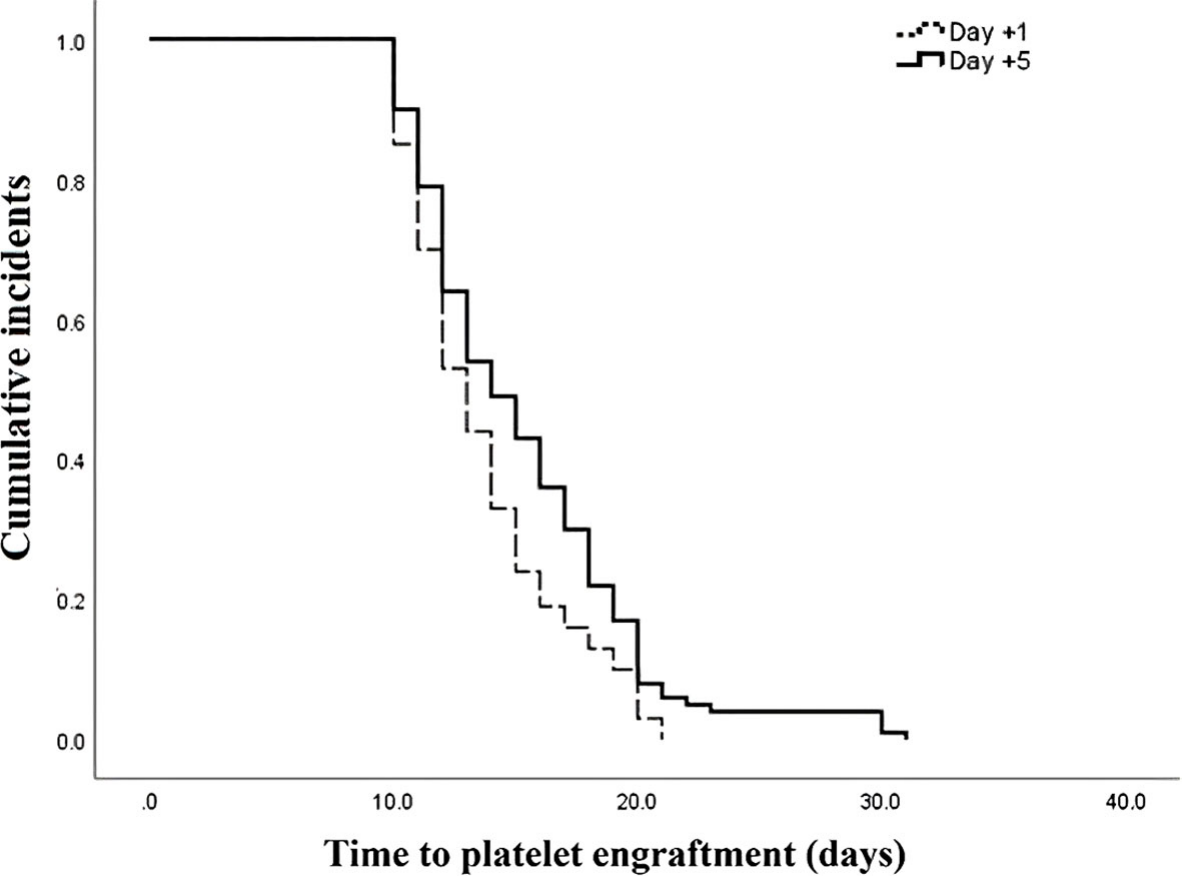
Time to platelet engraftment compared between patients with early (day +1) or delayed (day +5) initiation of granulocyte colony-stimulating factor (G-CSF) following autologous hematopoietic stem cell transplantation.

**Figure 2:**
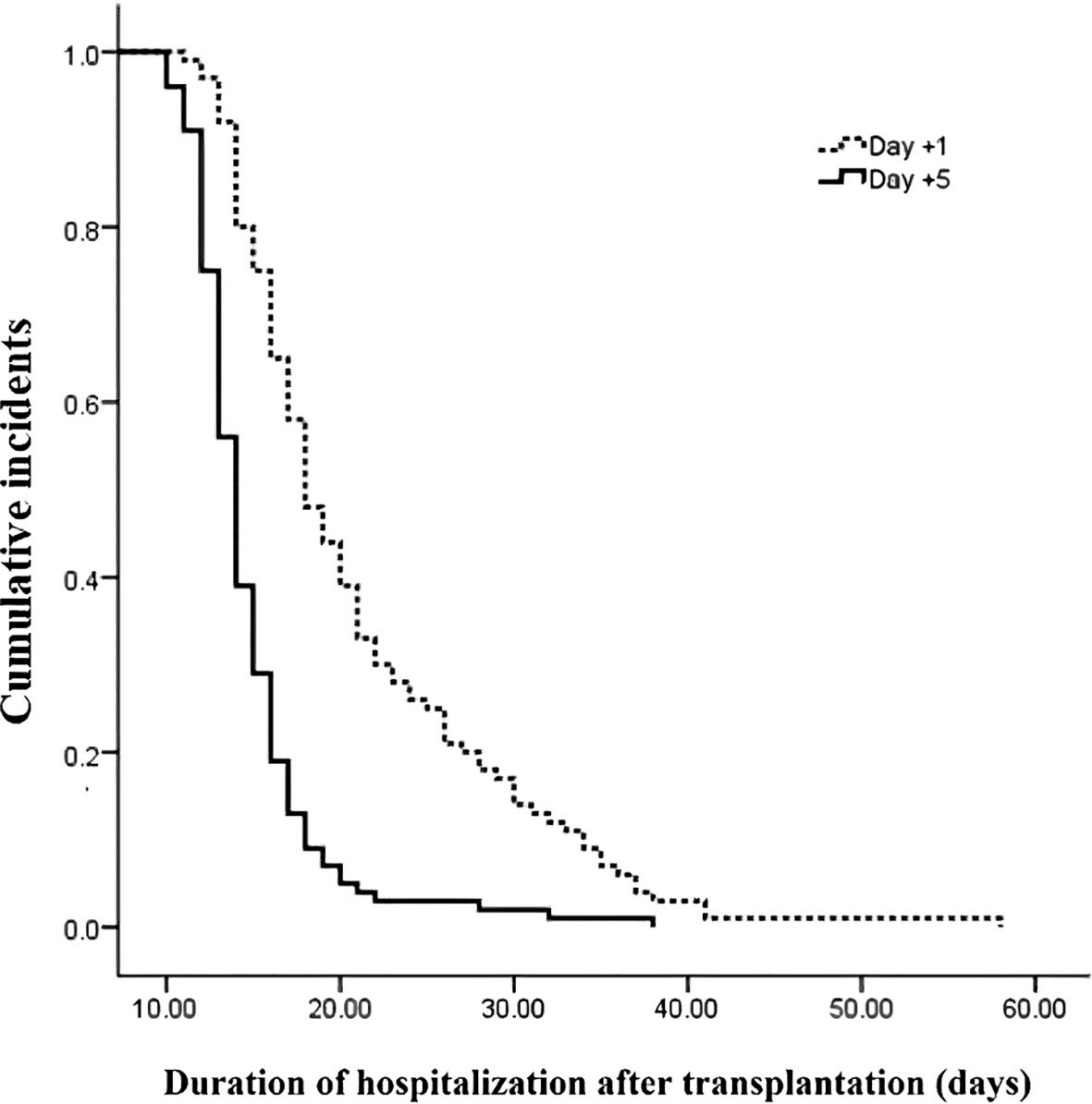
Duration of hospitalization after transplantation compared between patients with early (day +1) or delayed (day +5) initiation of granulocyte colony-stimulating factor (G-CSF) following autologous hematopoietic stem cell transplantation.

**Figure 3:**
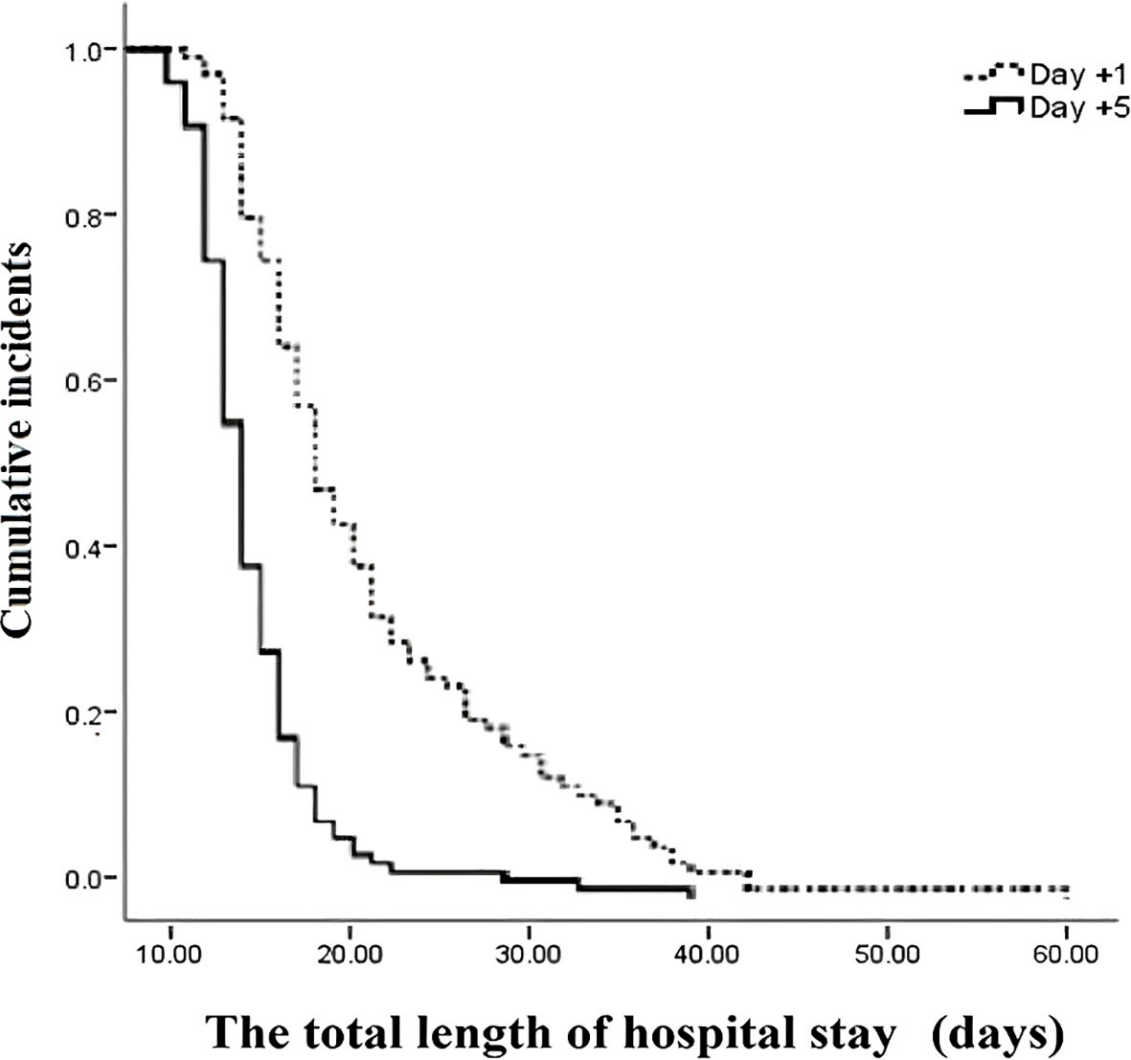
The total length of hospital stay compared between patients with early (day +1) or delayed (day +5) initiation of granulocyte colony-stimulating factor (G-CSF) following autologous hematopoietic stem cell transplantation.

**Figure 4:**
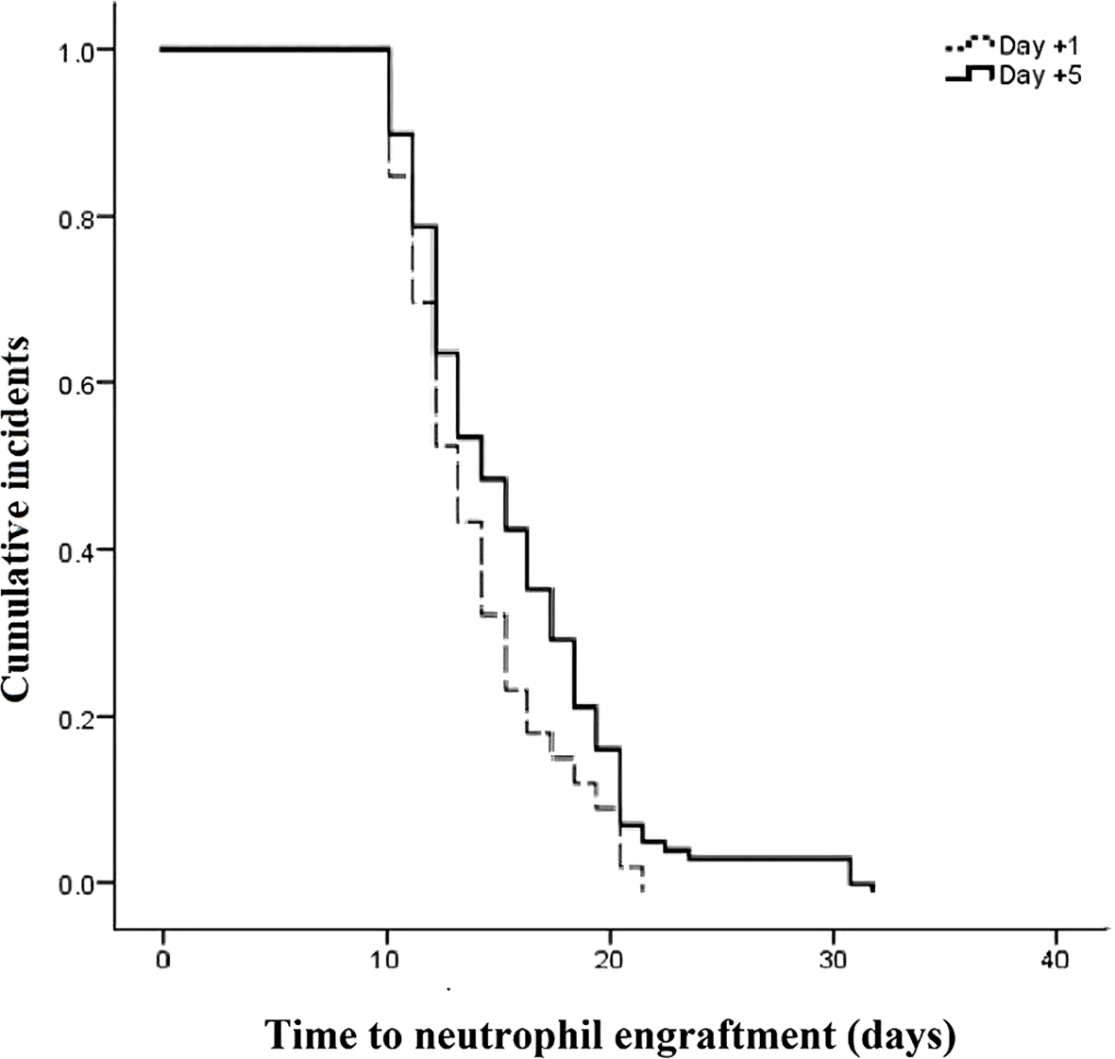
Time to neutrophil engraftment compared between patients with early (day +1) or delayed (day +5) initiation of granulocyte colony-stimulating factor (G-CSF) following autologous hematopoietic stem cell transplantation.

The outcomes of patients after receiving auto-HSCT are listed in **Table 2**. The total number of GCSF injections was significantly higher in the day +1 group compared with the day +5 group (*P*=0.005). The median time to platelet engraftment (**Figure 1**) was markedly higher among patients who received GCSF on day +1 vs. day +5 after transplantation (14 days *vs.* 12 days, *P*<0.001); the duration of hospitalization after transplantation (**Figure 2**) and the total length of hospital stay (**Figure 3**) were also significantly longer in the day +1 group (18 *vs.* 14 days, *P*<0.001 and 29 *vs.* 25 days, *P*<0.001, respectively). Time to neutrophil engraftment (**Figure 4**), duration of fever, bacteremia, fungal infection, and prophylactic drug use were comparable between the two groups (*P*>0.05) (**Table 2**).

**Table 2:** Clinical outcomes of patients based on time of granulocyte colony-stimulating factor (GCSF) initiation.

Outcomes	Day +1 group (N=100)	Day +5 group (N=100)	P-value
**Number of GCSF vial infusions, n** **Range** **Median (IQR)** **Mean ± SD**	7 – 31 14 (11.25 – 22) 14.67 ± 5.25	6 – 29 13 (11 – 16) 13.2 ± 5.21	0.005
**Time to neutrophil engraftment, days** **Range** **Median (IQR)** **Mean ± SD**	8 – 22 11 (11 – 12) 12.25 ± 2.8	8 – 21 11 (10 – 13) 11.62 ± 2.13	0.14
**Time to platelet engraftment, days** **Range** **Median (IQR)** **Mean ± SD**	6 – 25 14 (12 – 21) 14 ± 3.75	9 – 22 12 (11 – 14) 12.69 ± 2.4	<0.001
**Duration of fever, days** **Range** **Median (IQR)** **Mean ± SD**	0 – 12 2 (1 – 3) 2.27 ± 1.88	0 – 11 2 (1 – 3) 2.11 ± 1.83	0.13
**Duration of hospitalization after transplantation, days** **Range** **Median (IQR)** **Mean ± SD**	11 – 27 18 (15.25 – 25.75) 18 ± 3.83	10 – 22 14 (12.25 – 16) 14.2 ± 2.48	<0.001
**The total length of hospitalization, days** **Range** **Median (IQR)** **Mean ± SD**	20 – 38 29 (26 -35) 29 ± 4.43	17 – 40 25 (22 – 28) 25 ± 4.91	<0.001
**Bacteremia, n (%)**	32 (32.0%)	27 (27.0%)	0.46
**Fungal infection, n (%)**	6 (6.0%)	7 (7.0%)	0.76
**Pretransplantation myelotoxic drug use, n (%) ^1^** **Melphalan** **Procarbaribe** **Bendamustine** **Chlorambucil**	10 (10.0%) 6 (54.5%) 1 (9.1%) 3 (27.3%) 0 (0.0%)	5 (5.0%) 0 (0.0%) 0 (0.0%) 3 (60.0%) 2 (40.0%)	0.18
**Transfused pack cell units, n (mean)**	1.72	0.75	<0.001
**Transfused platelet units, n (mean)**	2.11	1.98	0.12

GCSF, granulocyte colony-stimulating factor; IQR, interquartile range; N, number; SD, standard deviation.

^1^Histories of myelotoxic drug use prior to transplantation, which may have contributed to delays in mobilization and engraftment.

## 4 Discussion

As a growing body of literature demonstrates, GCSF administration following hematopoietic stem cell transplantation is associated with reduced time for stem cell engraftment, decreased time of neutropenia, lower risk of infection, reduced length of hospital stay, and a lower mortality rate (12, 15–17). Although GCSF administration following HSCT is strongly recommended for better clinical outcomes, the optimal dosage and timing of GSCF initiation remain undetermined. We evaluated and compared clinical outcomes between patients who received early or delayed administration of GCSF following auto-HSCT; delayed initiation at day +5 after auto-HSCT was associated with earlier platelet engraftment, reduced packed cell, platelet, and GCSF administration, and reduced length of hospital stay compared to early initiation of GCSF treatment at day +1 post-transplantation. Moreover, the time interval for neutrophil engraftment, total days of febrile neutropenia, and infection rate were unaffected by delaying GCSF administration from day +1 to day +5 after auto-HSCT.

Several studies have been conducted to determine the optimal timing of GCSF initiation following stem cell transplantation (18–22); however, their results are conflicting. Following our findings, Demirer et al. revealed that time to neutrophil count recovery was not significantly different between patients who received GCSF at day 0 and those who received GCSF at day +5 following autologous stem cell transplantation (12). Moreover, delayed administration of GCSF from day 0 to day +5 following transplantation did not lead to an increased length of hospitalization, which is in line with our findings. Similarly, Janusek et al. (23) demonstrated that delaying the administration of GCSF until day +10 following autologous HSCT was not associated with deteriorated clinical outcomes relative to initiation at day +5 (23). Janusek et al. found that although delayed initiation of GCSF was associated with an increased time of neutrophil engraftment, the length of hospitalization was not affected (23). In our study, although treatment with GCSF at day +5 following autologous HSCT had no significant impact on the time of neutrophil engraftment, a decreased length of hospitalization was observed. These findings may indicate that the length of hospital stay is affected by neutrophil engraftment time, and other related factors like infections and platelet engraftment time may affect the clinical outcomes.

Our study revealed that patients who received GCSF at day +5 following auto-HSCT experienced faster platelet engraftment and a lower rate of transfusion of blood products than patients with an early administration of GCSF at day +1. In contrast to our findings, Demirer et al. showed that platelet recovery time, RBC transfusion, and platelet administration rate were comparable between patients who received GCSF at day 0 and day +5 following autologous stem cell transplantation (12). Ali et al. (24) also found that platelet recovery time was comparable between patients who received early GCSF at day 0 and those who underwent delayed GCSF administration at day +5 following auto-HSCT (24). Another recent study showed that time to platelet engraftment was significantly longer in patients who underwent delayed administration of GCSF at day +12 than those who experienced an early reception of GCSF at day +4 following transplantation (25). Considerable variations in study designs, participants, sample sizes, timings, and dosages of GCSF might be responsible for these differences.

A recent study found that delayed initiation of GCSF treatment at day +12 following stem cell transplantation was associated with lower total doses of GCSF (25), aligning with our finding that the total number of GCSF doses was significantly lower in patients who underwent delayed administration of GCSF at day +5 vs. day +1 after auto-HSCT. Similarly, Monge et al. (26) showed that in multiple myeloma patients undergoing autologous stem cell transplantation who received delayed GCSF administration on day +12 following transplantation, the total number of GCSF doses was lower relative to early treatment initiation at day +1 (26). Another study by Janusek et al. also found that significantly lower doses of GSCF were administered in patients who underwent delayed treatment of GCSF at day +5 following stem cell transplantation compared with those who received early GCSF administration (23). Moreover, in our study, the infection rate and the total number of febrile days were comparable between patients who received early and delayed GCSF, as observed in previous studies (12, 23–26). These findings indicate that delayed initiation of GCSF can be associated with reduced costs due to lower doses of injected GCSF, lower risk of infection, and decreased need to manage infectious diseases. Although our study did not evaluate hospitalization costs, we hypothesize that delayed administration of GCSF at day +5 following auto-HSCT might lead to a reduction in patient costs since the total doses of GCSF decreased, the infection rate remained unaffected, and the length of hospitalization fell, in line with prior studies (16, 19).

In contrast to our study, Sborov et al. (27) found that the duration of hospitalization and risk of infection was lower in multiple myeloma patients who underwent early GCSF administration at day +1 following autologous transplantation vs. those who received GCSF at day +5 or +7 after stem cell transplantation (27). Also, Hatch et al. (25) showed that patients undergoing auto-HSCT who received GCSF at day +4 following transplantation had faster neutrophil and platelet engraftment and shorter hospital stays when compared to those who received GCSF at day +12 after stem cell transplantation (25). Similarly, Monge et al. (26) demonstrated that delaying administration of GCSF from day +1 to day +12 after autologous stem cell transplantation in multiple myeloma patients led to increased neutrophil recovery time, neutropenia duration, and hospital stay (26), contrasting with our findings. One possible explanation for the existing differences is that in the earlier studies, patients underwent significantly delayed GCSF administration at day +12 following transplantation. Thus, although delayed administration of GCSF until day +5 following auto-HSCT may preserve the treatment efficacy, very delayed administration of GCSF is not recommended since it might be associated with an increased risk of developing unfavorable outcomes. Moreover, it is essential to note that our sample size was considerably larger than those of these two studies, each of which enrolled less than 100 patients. Nonetheless, further studies are needed to determine the optimal timing of GCSF initiation among patients undergoing auto-HSCT.

This study’s limitations include its single-center and cross-sectional design. We also enrolled patients with different underlying malignancies whose disease features might vary and affect the clinical outcomes. Finally, the potential effect of GCSF timing on hospitalization costs was not evaluated.

The present study has several strengths. First, a relatively large patient population was reviewed. Second, since the current study was performed at a single center, a uniform patient group was enrolled, lowering the risk of heterogeneity bias regarding the study population and treatment modalities. Third, data regarding patient characteristics and outcomes were all computerized which reduced the amount of missing data. However, the present study has also several limits which need to be acknowledged. The study was retrospective in design, introducing some degrees of restriction and bias risk. Moreover, the effect of various confounders was not excluded. Although several studies have been conducted, the optimal timing of GCSF initiation in cancer patients undergoing hematopoietic stem cell transplantation remains unclear since the literature is heterogeneous. Thus, multi-center studies with large sample sizes and randomized controlled designs are needed to elucidate the best timing of GCSF initiation among patients with malignancy who undergo auto-HSCT. In addition, evaluation of other clinical outcomes is strongly recommended.

## 5 Conclusion

The current study revealed that delayed administration of GCSF at day +5 following transplantation in patients with hematological or solid malignancies was associated with earlier platelet recovery, reduced need for blood product transfusion, reduced GCSF administration doses, and decreased hospitalization length. Moreover, no adverse effects regarding neutrophil engraftment time, febrile neutropenia duration, or rate of developing infectious diseases were found when delaying GCSF administration from day +1 to day +5 after auto-HSCT. The present study’s findings suggest that delayed administration of GCSF at day +5 following auto-HSCT can be associated with favorable clinical outcomes, equal treatment efficacy, and reduced hospitalization costs. However, the optimal timing of GCSF treatment initiation in patients receiving stem cell transplantation is yet to be understood. Hence, further studies are needed to examine the optimal timing of GCSF administration and related clinical outcomes in patients with malignancy who undergo stem cell transplantation.

## Data Availability

The raw data supporting the conclusions of this article will be made available by the authors, without undue reservation.
